# TLR4-RelA-miR-30a signal pathway regulates Th17 differentiation during experimental autoimmune encephalomyelitis development

**DOI:** 10.1186/s12974-019-1579-0

**Published:** 2019-09-27

**Authors:** Xuebin Qu, Jingjing Han, Ying Zhang, Xingqi Wang, Hongbin Fan, Fang Hua, Ruiqin Yao

**Affiliations:** 1 Department of Cell Biology and Neurobiology, Xuzhou Key Laboratory of Neurobiology, Xuzhou Medical University, Xuzhou, 221009 Jiangsu People’s Republic of China; 2 Department of Neurology, Affiliated Hospital of Xuzhou Medical University, Xuzhou, 221002 Jiangsu China; 3 School of Life Science, Jiangsu Normal University, Xuzhou, 221116 Jiangsu China; 4 Institute of Neurological Diseases of Xuzhou Medical University, Xuzhou, 221002 Jiangsu China

**Keywords:** Multiple sclerosis, T helper cell, Differentiation, Neuroinflammation, TLR4, RelA, MiR-30a

## Abstract

**Background:**

Toll-like receptor 4 (TLR4) is well known for activating the innate immune system; however, it is also highly expressed in adaptive immune cells, such as CD4^+^ T-helper 17 (Th17) cells, which play a key role in multiple sclerosis (MS) pathology. However, the function and governing mechanism of TLR4 in Th17 remain unclear.

**Methods:**

The changes of TLR4 in CD4^+^ T cells from MS patients and experimental autoimmune encephalomyelitis (EAE) mice were tested. TLR4-deficient (TLR4^−/−^) naïve T cells were induced in vitro and transferred into Rag1^−/−^ mice to measure Th17 differentiation and EAE pathology. DNA sequence analyses combining with deletion fragments and mutation analyses, chromatin immunoprecipitation (ChIP), and electrophoretic mobility shift assay (EMSA) were used to explore the mechanism of TLR4 signaling pathway in regulating Th17 differentiation.

**Results:**

The levels of TLR4 were increased in CD4^+^ Th17 cells both from MS patients and EAE mice, as well as during Th17 differentiation in vitro. TLR4^−/−^ CD4^+^ naïve T cells inhibited their differentiation into Th17, and transfer of TLR4^−/−^ CD4^+^ naïve T cells into Rag1^−/−^ mice was defective in promoting EAE, characterized by less demyelination and Th17 infiltration in the spinal cord. TLR4 signal enhanced Th17 differentiation by activating RelA, downregulating the expression of miR-30a, a negative regulator of Th17 differentiation. Inhibition of RelA activity increased miR-30a level, but decreased Th17 differentiation rate. Furthermore, RelA directly regulated the expression of miR-30a via specific binding to a conserved element of miR-30a gene.

**Conclusions:**

TLR4^−/−^ CD4^+^ naïve T cells are inadequate in differentiating to Th17 cells both in vitro and in vivo. TLR4-RelA-miR-30a signal pathway regulates Th17 differentiation via direct binding of RelA to the regulatory element of miR-30a gene. Our results indicate modulating TLR4-RelA-miR-30a signal in Th17 may be a therapeutic target for Th17-mediated neurodegeneration in neuroinflammatory diseases.

## Background

Multiple sclerosis (MS) is an inflammatory demyelinating syndrome of the central nervous system (CNS) that is characterized by progressive immune-mediated destruction of the myelin sheath and accumulated neurological disability [1, 2]. Main contributors to myelin sheath damage are activated myelin-reactive T cells that infiltrate the CNS, triggering an immunologic cascade [3, 4]. In MS and an experimental autoimmune encephalomyelitis (EAE) mouse model, mature and excessively activated IL-17-secreting T helper cells 17 (Th17) are major initiators and participants involved in promoting pathology. Infusion of Th17 cells or injection of IL-17 can effectively aggravate disease, while IL-17-deficient mice have alleviated pathology [5]. In addition, it has been shown that the pathogenicity of autoreactive Th17 in mice is linked with their production of GM-CSF [6–8]. Thus, downregulation of the immune response, especially Th17 cell differentiation and activation, may be an effective treatment strategy for MS.

Toll-like receptors (TLRs) are important innate immune proteins that play critical roles in initiating inflammatory responses and promoting adaptive immune responses responsible for the identification and clearance of invading pathogens [9]. Inappropriate activation of TLRs, such as TLR4, and downstream pathways have been implicated in certain autoimmune diseases, including MS [10]. TLR4 is upregulated in patients with MS and in the spinal cord of EAE mice [1, 11–13]. In vivo, treatment with TLR4 ligands, such as LPS, aggravates EAE, while targeted disruption of TLR4 prevents EAE [14–19]. Activation of TLR4 produces high levels of proinflammatory cytokines including IL-1β and IL-6 [20, 21], which initiate differentiation of Th17 cells. Activated Th17 cells can secrete IL-17 and GM-CSF [6, 7, 22, 23], resulting in a CNS autoimmunity for demyelination. Besides innate immune cells such as microglia and macrophages, TLR4 is also expressed in a wide range of adaptive immune cells including CD4^+^ T cells, even higher in Th17 cells than in Th1 and Th2 cells [24, 25]. However, little is known about the role of TLR4 activation in Th17.

It has been shown that TLR4^−/−^ CD4^+^ T cells almost completely abrogate EAE symptoms through blunted Th17 activation and defective IL-17 production [25]. Consistently, CD4^+^ naïve T cells stimulated through the TLR4 pathway proliferate more extensively and exhibit enhanced survival in vitro [25]. Although TLR4 activation plays a role in Th17 proliferation, several questions remain unanswered, including (1) the inconsistent role of TLR4 activation on Th17 differentiation in vitro and in vivo and (2) the molecular mechanisms that govern Th17 differentiation following TLR4 stimulation in CD4^+^ T cells.

MicroRNAs (miRNAs) have been established as powerful regulators of gene expression in normal physiological, as well as in pathological, conditions. Also, miRNAs can serve as diagnostic biomarkers and therapeutic targets for many diseases [26, 27]. Many researchers have identified major factors participating in miRNA biogenesis and established basic principles of miRNAs function [28], but the upstream transcriptional regulation of these miRNAs is not well understood. Limited studies show that miRNA expression can be directly regulated by epigenetic alterations, such as DNA methylation [29–31] and histone modification [32, 33]. In addition, transcription factors, such as NF-κB, MYC, REST, and STAT, are thought to regulate miRNA transcription in a similar manner to that of protein-coding genes by binding to conventional DNA binding sequences located in or near regulatory regions that lie upstream of microRNA genes [28, 34–37]. Based on our earlier data, the disordered miR-30a level in vivo contributes to Th17 ratio imbalance and EAE pathology [38]; however, the reasons for dysregulated expression of miR-30a in EAE mice and MS patients are unclear. Interestingly, by bioinformatic analyses, we find several potential binding sites of RelA, a nuclear transcription factor that is activated by TLR4, in the promoter region of miR-30a gene, suggesting a possible regulating role of TLR4-RelA on miR-30a expression.

In this paper, our results demonstrate that TLR4^−/−^ CD4^+^ naïve T cells are inadequate in differentiating to Th17 cells both in vitro and in vivo. Furthermore, TLR4-RelA-miR-30a signal pathway regulates Th17 differentiation via direct binding of RelA to the regulatory element of miR-30a gene.

## Materials and methods

### Clinical subjects

MS patients (Additional file 1: Table S1) were recruited in Affiliated Hospital of Xuzhou Medical University and Xuzhou Central Hospital. All participants were examined by neurologists for expanded disability status scale measures [39]. Peripheral blood samples were obtained from MS patients as well as age- and sex-matched healthy volunteers. Written informed consents were obtained from all of the participants. Research protocols were approved by the appropriate institution review boards.

### Mice and EAE induction

C57BL/6 wild-type (WT) mice were purchased from SLAC Laboratory Animal Co., Ltd. (Shanghai, China). TLR4^−/−^ and Rag1^−/−^ mice were from the Model Animal Research Center of Nanjing University (Nanjing, China). All mice were housed under specific pathogen-free conditions in the Xuzhou Medical University animal facility (Xuzhou, China). All experiments were performed in accordance with the Provisions and General Recommendations of the Chinese Experimental Animal Administration Legislation, as well as institutional approval from the Xuzhou Medical University Experimental Animal Ethics Committee.

The EAE model was performed as described previously [38]. Briefly, female mice 7–8 weeks old were immunized subcutaneously with 100 μg MOG 35–55 (MEVGWYRSPFSRVVHLYRNGK) (GL Biochem, Shanghai, China) in complete Freund’s adjuvant (Sigma-Aldrich, St. Louis, MO, USA) containing 4 mg/mL heat-killed *Mycobacterium tuberculosis* H37Ra (BD Bioscience, San Jose, CA, USA). Each mouse was administered intraperitoneally (i.p.) with 200 ng pertussis toxin (Sigma-Aldrich, USA) twice, on the day of immunization and 48 h later. All of the mice were weighted daily, and the ratio of body weight was normalized to the initial weight of each mouse. Clinical assessment of EAE was performed daily according to the following criteria: 0, no clinical signs; 1, paralyzed tail; 2, paresis (weakness, incomplete paralysis of one or two hindlimbs); 3, paraplegia (complete paralysis of both hindlimbs); 4, paraplegia with forelimb weakness or paralysis; and 5, moribund state or death.

### CD4^+^ naïve T cell culture, purification, transfer, and induction

CD4^+^ naïve T cells from splenocytes of 5- to 6-week-old mice were purified by magnetic cell sorting according to the manufacturer’s instructions (Miltenyi Biotec, Bergisch Gladbach, Germany). Briefly, non-naïve CD4^+^ T cells were indirectly magnetically labeled by using a cocktail of biotin-conjugated antibodies and anti-biotin microbeads. Memory T cells were directly magnetically labeled with CD44 microbeads. Isolation of pure naïve T cells was achieved by depletion of magnetically labeled non-target cells. For adoptive transfer studies, each recipient Rag1^−/−^ mouse was injected intravenously in tail veins with 5 × 10^6^ CD4^+^ naïve T cells on the day before EAE immunization.

For Th17 differentiation, the purified CD4^+^ naïve T cells were cultured for 3 days under Th17 cell-polarizing conditions (RPMI-1640 containing 10% FBS, 1 mM glutamine, 0.1 mM b-mercaptoethanol, 1% nonessential amino acids (Sigma–Aldrich, USA), anti-CD3 plus anti-CD28-coated beads (Invitrogen, CA, USA), 5 ng/mL IL-2 (R&D Systems Inc., Minneapolis, MN, USA), 20 ng/mL IL-6, 5 ng/mL transforming growth factor-β, 10 ng/mL IL-23, 2 μg/mL anti-IL-4, and 2 μg/mL anti-interferon-γ (BD Bioscience, USA). In some experiments, 10 μg/ml LPS (Sigma-Aldrich, USA), 5 μM TPCA-1 (Abcam, Cambridge, UK), or 10 μM Helenalin (Abcam, UK) were used.

To enrich IL-17^+^ Th17 [38], cells were incubated with Cell Stimulation Cocktail (eBioscience, CA, USA) for 5 h, and then purified by magnetic cell sorting according to manufacturer’s instructions (Miltenyi Biotec, Germany): briefly, an IL-17-specific catch reagent was attached to the cell surface to bind with the secreted IL-17 from positive, secreting cells. These cells were subsequently labeled with a second IL-17-specific antibody conjugated to biotin, and then incubated with anti-biotin antibody conjugated to R-phycoerythrin (PE). Then, the IL-17-secreting cells were magnetically labeled with anti-PE microbeads and enriched in the magnetic field.

### Histological analyses

After anesthesia with pentobarbital, mice were perfused with normal saline and then buffered 4% paraformaldehyde. Following standard protocols, paraffin-embedded sections of spinal cords were stained with hematoxylin and eosin (H&E) or luxol fast blue (LFB) for analysis of inflammation or demyelination, respectively. For H&E staining, sections were deparaffinized, rehydrated, and stained in hematoxylin solution for 8 min. Then, the sections were rinsed in distilled water and 95% ethyl alcohol, followed by counterstain in eosin solution for 1 min. After dehydrated through ethyl alcohol and cleared in xylene, the stained sections were mounted for observation. Spinal cord infiltrates were quantified as infiltrates per square millimeter in five non-serial H&E-stained sections from each mouse in a blinded manner. For LFB staining, the deparaffinized sections were hydrated in 95% ethyl alcohol, then stained in luxol fast blue solution at 56 °C overnight. After rinse in 95% ethyl alcohol and distilled water, sections were differentiated with lithium carbonate solution and 70% ethyl alcohol. Then, the sections were rinsed, dehydrated, cleared, and mounted for observation. Demyelination was quantified by calculation of regional mean optical density (MOD) relative to the total value in five non-serial LFB-stained sections from each mouse in a blinded manner.

For immunofluorescence staining, prepared spinal cords were incubated overnight in sodium phosphate buffer containing 30% sucrose, then embedded in Optimal Cutting Temperature medium (Leica, Solms, Germany) for sectioning. Cryosections (15 μm) were thawed, washed with 5% normal goat serum and 0.3% Triton X-100 in 0.01% PBS, and then incubated with primary monoclonal antibody (CST, MA, USA) overnight at 4 °C. Following an additional wash step, specimens were incubated with goat anti-mouse or goat anti-rabbit fluorescently conjugated secondary antibodies (Santa Cruz, CA, USA), and cell nucleus was counterstained with 4′,6-diamidino-2-phenylindole (Vicmed, Xuzhou, China). Images were acquired using a fluorescence microscope system (Olympus, Tokyo, Japan), and MFI values were calculated using ImageJ software.

### Quantitative RT-PCR

Cells were lysed by TRIzol (Invitrogen, USA) for RNA isolation. One microgram of total RNA from each sample was reverse-transcribed using a QuantScript RT kit (Tiangen, Beijing, China) and examined with a SYBR Green real-time PCR kit (Roche, Basel, Switzerland). To test the miR-30a expression, 500 ng total RNA from each sample was performed using a MiRcute MiRNA Kit (Tiangen, China). Relative expression of mRNA or miRNA was evaluated by the 2^−ΔΔCt^ method in LightCycler® 480ӀӀ System (Roche, Switzerland) and normalized to the expression of β-actin or U6 respectively. The primers were listed in Additional file 2: Table S2.

### Western blotting

Tissues or cells were stripped and ultrasonically homogenized in RIPA buffer, then quantified by a bicinchoninic acid protein assay kit (Beyotime, Shanghai, China). Protein samples were separated by electrophoresis in an SDS denaturing 10% polyacrylamide gels and transferred to nitrocellulose membranes. Membranes were blocked in 0.01% PBS containing 5% BSA, incubated overnight at 4 °C with primary antibodies, and then incubated in IRDye-conjugated secondary antibodies (LI-COR, CA, USA). Membranes were scanned using an Odyssey Infrared Imaging System Scanner (LI-COR, USA), and images were analyzed using ImageJ software.

### ELISA

Quantikine ELISA kits to measure IL-17A, IL-17F, and GM-CSF concentration were obtained from Westang Biological Technology Co., Ltd. (Shanghai, China) and used according to the manufacturer’s instructions. All samples were measured in duplicate.

### Flow cytometric analyses

Whole blood from clinical participant was collected by venipuncture in heparinized tubes and layered over Human Lymphocyte Separation Medium (Dakewe, Shenzhen, China) to obtain peripheral blood lymphocytes. Then, fluorescent-labeled antibodies, such as anti-CD4, CD45RA, or CD45RO (Miltenyi Biotec, Germany) were added and incubated for 30 min at 4 °C. For CD4^+^ IL-17^+^ T cell isolation, the peripheral blood lymphocytes were incubated with Cell Stimulation Cocktail (eBioscience, USA) for 5 h before CD4 staining, and then resuspended in fixation/permeabilization solution (BD Pharmingen, USA), after which anti-IL-17 fluorescent-labeled antibody (Miltenyi Biotec, Germany) was added and incubated for 30 min at room temperature. Finally, the labeled cells were acquired by flow cytometer (BD Biosciences, USA).

Peripheral blood, splenocytes, lymph nodes, and type I collagenase digested spinal cords from mice were collected; then, lymphocytes were prepared by Percoll gradient centrifugation. To detect intracellular cytokine levels, cells were incubated with Cell Stimulation Cocktail (eBioscience, USA) for 5 h, then cells were resuspended in fixation/permeabilization solution (BD Pharmingen, USA) and stained with anti-IL-17 and GM-CSF antibodies (Miltenyi Biotec, Germany) according to the manufacturer’s protocol. In some experiments, staining with anti-RelA antibody (CST, USA) and APC-conjugated secondary antibody (Miltenyi Biotec, Germany) was performed. Tests were performed on MACSQuantTM Flow Cytometers (Miltenyi Biotec, Germany) and analyzed with FlowJo software. Protein expression level was expressed as mean fluorescence intensity (MFI).

### DNA sequence analyses

Mmu-miR-30a precursor (MI0000144) DNA sequence (chr1:23272269-23272339), 5 kb spanning sequence upstream, and 1 kb spanning sequence downstream of transcription start site were obtained from miRBase (http://www.mirbase.org/) [40] and UCSC (http://genome.ucsc.edu/ENCODE/) [41]. Highly conservative transcription factor binding site clusters across three closely related mammals: mouse, human, and chimp, were aligned by ECR Browser (https://ecrbrowser.dcode.org/) [42] according to website instructions. Transcription factor binding sites were predicted using the JASPAR open-access database (jaspar.genereg.net/) [43] and rVista 2.0 online database (https://rvista.dcode.org/) [44] following website instructions.

### Plasmid construction and dual-luciferase reporter assay

Five regulatory element deletion fragments were amplified using primers (Additional file 3: Table S3). The purified PCR products were digested with *Kpn*I and *Xho*I and ligated into the pGL4.20[luc2Puro] vector (Promega, WI, USA). For the binding site mutation assay, synthesized DNA sequences containing mutated binding sites were cloned into the pGL4.20[luc2Puro] vector.

Recombinant plasmids and internal control vector PRL-TK Renilla vector were transfected into *Jurkat cells* using Lipofectamine 2000 reagent (Invitrogen, USA) following the instructions. Cells were harvested at 48 h post-transfection and assayed for luciferase activity using the Dual-Luciferase Reporter Assay System (Promega, USA).

### Electrophoretic mobility shift assays

Cellular nuclear protein was extracted with Nucleoprotein Extraction Kit (Beyotime, China). Double-stranded oligonucleotides (Sangon, Shanghai, China) corresponding to the RelA binding sites of the mmu-miR-30a precursor regulatory elements were synthesized and annealed into double strands. The probes are listed in Additional file 4: Table S4.

A total of 12 μg nuclear extract was added to 0.1 μM biotin-labeled double-stranded oligonucleotides in 1×EMSA/Gel-Shift binding buffer, while extra 5 μM unlabeled competitor oligonucleotide was added in the control group and 2 μg of RelA antibody was needed for the super-shift reaction. Mixtures were incubated at 24 °C for 20 min, analyzed by electrophoresis in 4% polyacrylamide gels at 10 V/cm, and then transferred to a nylon membrane. Membranes were UV-light cross-link, incubated with HRP-conjugated Streptavidin, and developed with chemiluminescences.

### Chromatin immunoprecipitation assay

ChIP assays were performed using an EZ-ChIP™ kit (Millipore, MA, USA) according to the manufacturer’s instructions. Briefly, chromatin was crosslinked with 1% formaldehyde at 37 °C for 10 min and reactions were neutralized with glycine for 5 min at room temperature. Cells were then harvested, lysed, and sonicated. Nuclear lysates were sonicated 15 times for 4.5 s each with 9 s intervals on ice water using a Scientz-IID (Scientz, Zhejiang, China). After incubation with Protein A Agarose/Salmon Sperm DNA at 37 °C for 1 h, an equal amount of chromatin was immuno-precipitated at 4 °C overnight with at least 1 μg of RelA (Abcam, UK) or isotype IgG antibodies. Immuno-precipitated products were collected after incubation with Protein A Agarose/Salmon Sperm DNA, and then, the bound chromatin was eluted in ChIP elution buffer. The proteins were digested with Proteinase K for 2 h at 45 °C, and the DNA was purified for PCR test. Primers for PCR were listed in Additional file 5: Figure S1.

### Statistical analyses

The results were expressed as mean ± standard deviation (SD) and analyzed by SPSS 17.0. Independent sample *t* tests were used to evaluate the differences between groups. One-way analysis of variance (ANOVA) followed by Bonferroni’s post hoc test was used for multiple comparisons. Body weights and clinical scores were evaluated by two-way repeated measures ANOVA. A *P* value of 0.05 or less was considered significant.

## Results

### TLR4 is increased in Th17 during EAE and MS development

**Fig. 1:**
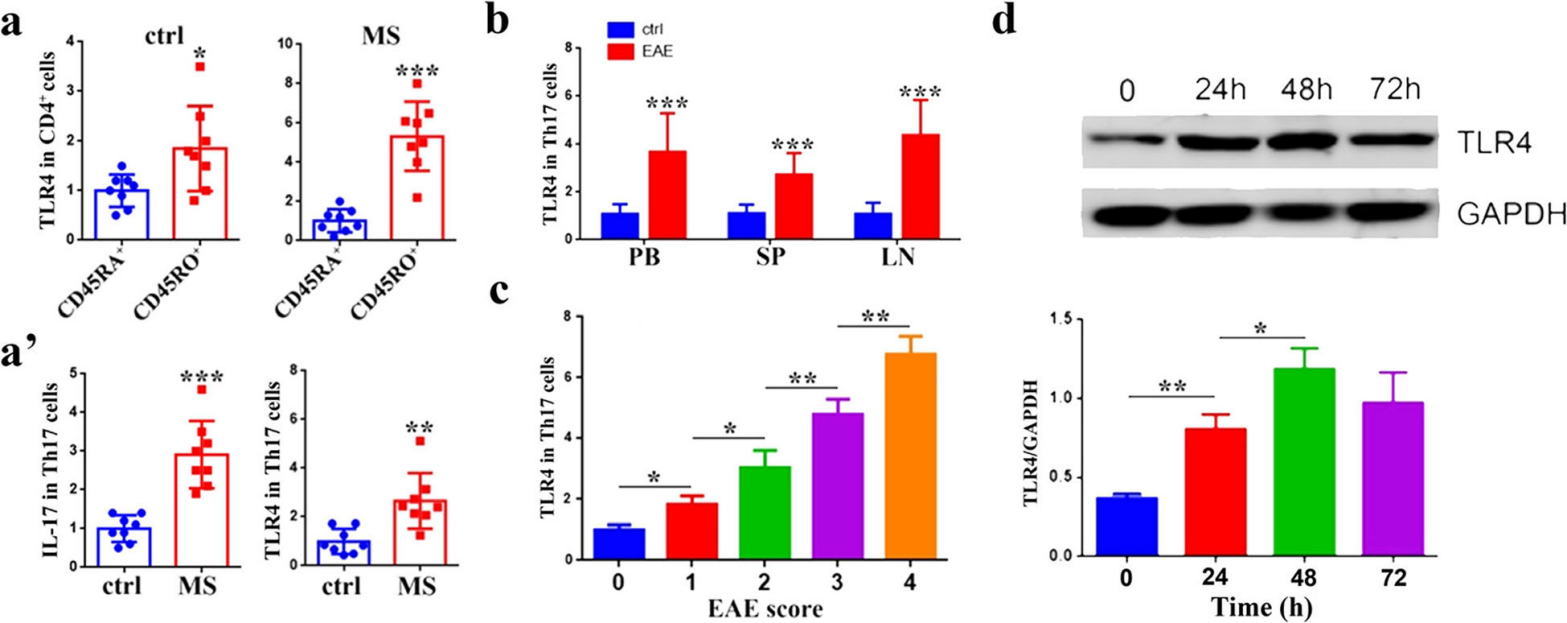
TLR4 is upregulated in Th17 cells from MS patients and EAE mice. **a** Quantitative PCR analyses of TLR4 expression in CD4^+^ peripheral blood lymphocytes between CD45RA^+^ naïve cells and CD45RO^+^ memory cells from healthy volunteers (ctrl) and MS patients (*n* = 8 per group). **a’** Quantitative PCR analyses of IL-17 and TLR4 expression in Th17 cells from peripheral blood lymphocytes between healthy volunteers (ctrl) and MS patients (*n* = 8 per group). **b** Quantitative PCR analyses of TLR4 expression in Th17 cells from peripheral blood (PB), splenocytes (SP), and lymph nodes (LN) between normal mice (ctrl) and EAE mice (*n* = 10 per group) 20 days after immunization. **c** The relative expression of TLR4 in Th17 cells from peripheral blood lymphocytes of EAE mice at different scores (*n* = 6 per group). **d** Western blot and quantification analyses of TLR4 expression during Th17 differentiation in vitro. Data are presented as mean ± standard deviation. *P < 0.05. **P < 0.01. ***P < 0.001; Student’s *t* test (**a**, **b**), one-way ANOVA (**c**, **d**). Data are representative of three experiments done in triplicate

We collected peripheral blood CD4^+^ T cells from MS patients and healthy controls and found MS patients had a more significantly upregulated expression of TLR4 (> 5 folds) in CD45RO^+^ memory CD4^+^ T cells than CD45RA^+^ naïve T cells compared with that in healthy controls (< 2 folds) (Fig. 1a). As Th17 cells are key participants involved in MS development, we enriched IL-17^+^ Th17 cells and found Th17 cells from MS patients expressed more IL-17 and TLR4 than those from healthy controls (Fig. 1a’). Similarly, in EAE mice, TLR4 was also increased dramatically in Th17 cells from peripheral blood, spleen, and draining lymph nodes (Fig. 1b). Moreover, as EAE was more aggravated (i.e., score increased), the level of TLR4 was correspondingly increased (Fig. 1c). To further explore the relationship between TLR4 expression and Th17 cells, we induced CD4^+^ naïve T cells into Th17 in vitro and found that TLR4 had a gradually ascending pattern with reaching its peak at 48 h, then kept a high expression until 72 h (Fig. 1d). These phenomena suggest that TLR4 in CD4^+^ T cells may play a role in Th17 differentiation and MS development.

### TLR4 deficiency inhibits the generation of Th17 cell in vitro

**Fig. 2:**
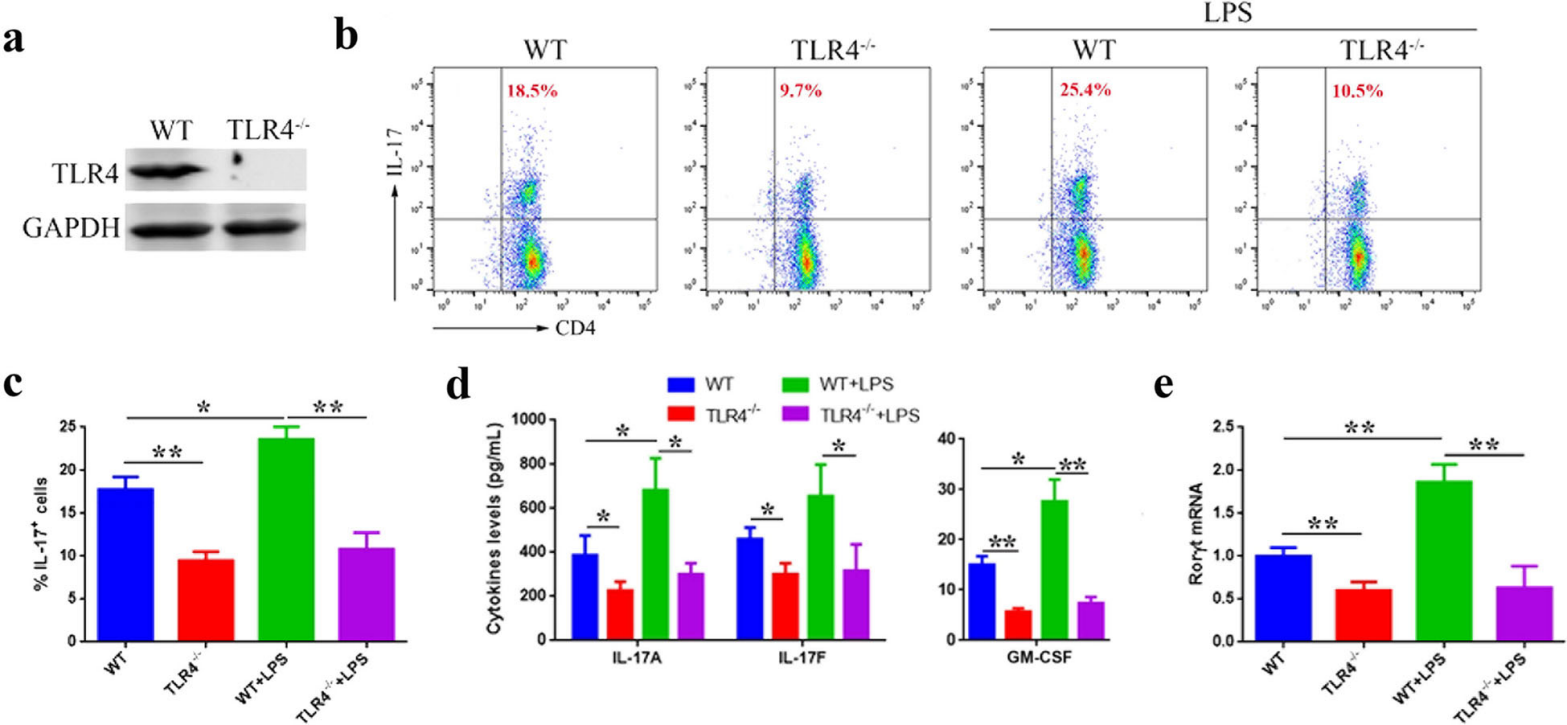
TLR4 deficiency suppresses Th17 differentiation in vitro. CD4^+^ naïve T cells from TLR4^−/−^ or wild type (WT) mice are cultured in Th17-polarizing conditions with or without LPS for 3 days. **a** Western blot test of TLR4 in CD4^+^ naïve T cells from TLR4^−/−^ and WT mice. **b**, **c** Flow cytometric analyses of stimulated IL-17^+^ cells. **d** ELISA of IL-17A, IL-17F, and GM-CSF in culture supernatants. **e** Quantitative PCR analyses of Rorγt expression in cultured cells. Data are presented as mean ± standard deviation. *P < 0.05. **P < 0.01; one-way ANOVA. Data are representative of three experiments done in triplicate

To determine the role of TLR4 in Th17 differentiation, CD4^+^ naïve T cells from TLR4^−/−^ mice (Fig. 2a) were cultured under Th17-polarizing conditions with or without LPS. Data showed that TLR4 deficiency led to a lower percentage of induced IL-17^+^ cells, even LPS, which is an activator of Th17 differentiation, could not increase the IL-17^+^ cells in the absence of TLR4 (Fig. 2b, c). Secretion of the Th17-related cytokines, IL-17A, IL-17F, and GM-CSF (Fig. 2d), as well as expression of the lineage marker gene, Rorγt (Fig. 2e), was significantly downregulated in TLR4^−/−^ T cells, even in the presence of LPS. These results indicate that TLR4 contributes to Th17 differentiation.

### TLR4^−/−^ CD4^+^ naïve T cells are defective in promoting EAE

**Fig. 3:**
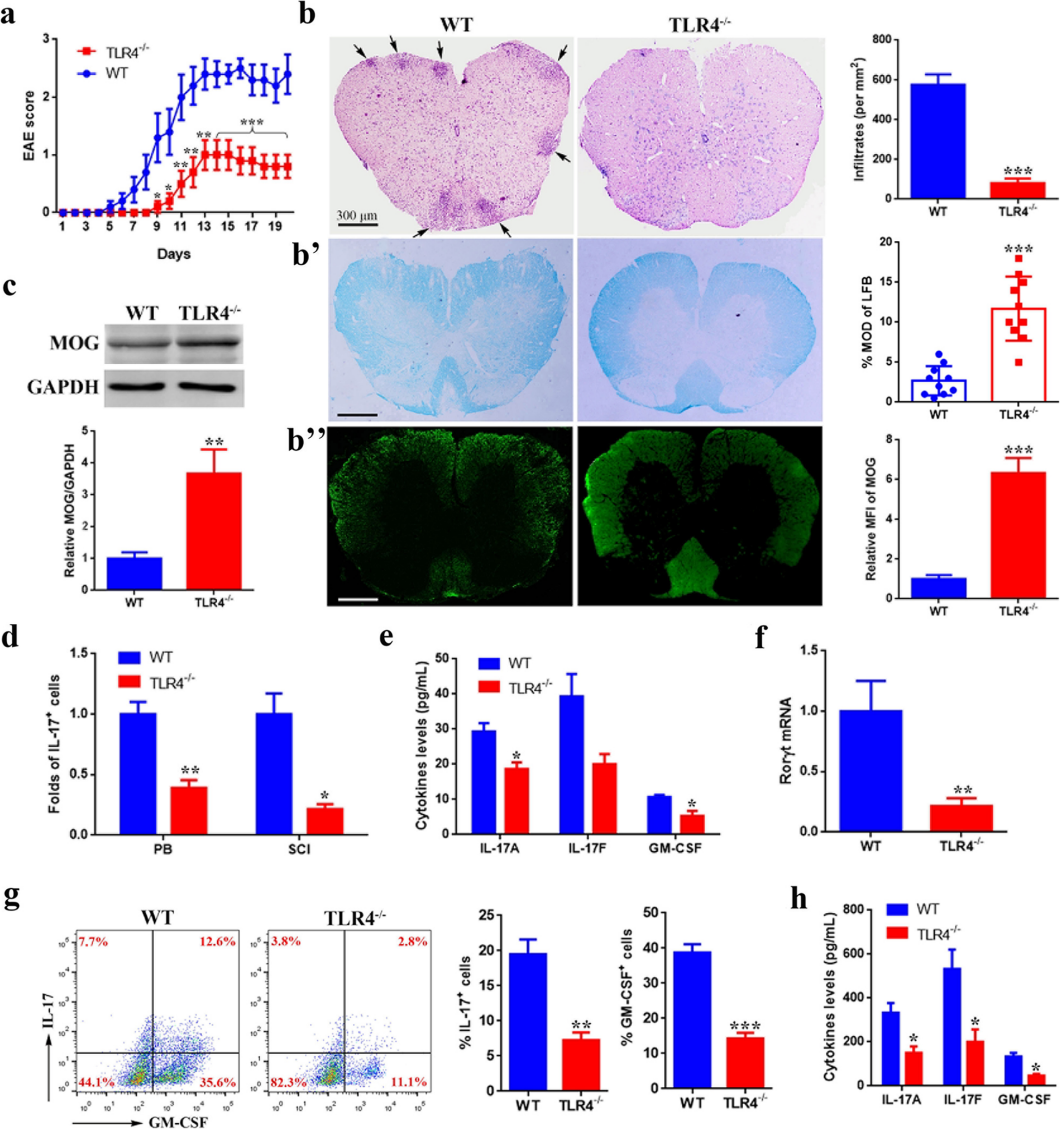
TLR4^−/−^ CD4^+^ naïve T cells are defective in promoting EAE. Rag1^−/−^ mice transplanted with wild type (WT) or TLR4^−/−^ CD4^+^ naïve T cells are immunized with MOG for EAE induction. **a** Mean EAE scores of mice (*n* = 5 per group). **b**–**b”** H&E staining (**b**), LFB staining (**b’**), and MOG immunofluorescence staining (**b”**) of spinal cord sections from transplanted Rag1^−/−^ mice. Arrows show lymphocyte infiltration. Scale bar, 300 μm. Quantification analyses are on the right. **c** Western blot and quantification analyses of MOG expression in spinal cord. **d** Quantification analyses of IL-17^+^ cells among CD4^+^ gated cells from peripheral blood (PB) and spinal cord-infiltrated (SCI) lymphocytes by flow cytometry. **e** ELISA of IL-17A, IL-17F, and GM-CSF in peripheral blood serum. **f** Quantitative PCR analyses of Rorγt expression in spinal cord-infiltrated lymphocytes. **g**, **h** Splenocytes and lymph node cells from mice presented (**a**) on day 20 are stimulated in vitro. Flow cytometric analyses of IL-17^+^ and GM-CSF^+^ cells among CD4^+^ gated cells (**g**). The concentrations of IL-17A, IL-17F, and GM-CSF in supernatant are measured by ELISA (**h**). Data are presented as mean ± standard deviation. *P < 0.05. **P < 0.01. ***P < 0.001; two-way repeated measures ANOVA (**a**), Student’s *t* test (**b**–**h**). Data are representative of three experiments done in triplicate

To further clarify the role of TLR4 in the generation of Th17 cells in vivo, we transferred TLR4^−/−^ CD4^+^ naïve T cells into age-matched Rag1^−/−^ mice, which were then induced for EAE. In sharp contrast with control, mice receiving TLR4^−/−^ CD4^+^ naïve T cells had a less weight loss (Additional file 6: Figure S2), exhibited a lower EAE incidence, and developed a delayed onset with reduced EAE severity (Table 1, Fig. 3a). Histological analyses of spinal cord sections showed that TLR4^−/−^ CD4^+^ naïve T cell-transferred mice had minimal inflammatory infiltration (Fig. 3b) and demyelination (Fig. 3b’), which was further supported by the result of increased MOG (Figs. 3b”, c), a protein involved in structural integrity to the myelin sheath. Compared with the WT group, the proportions of IL-17^+^ cells in the peripheral blood and spinal cord-infiltrated lymphocytes were much lower in TLR4^−/−^ CD4^+^ naïve T cell-transferred mice (Fig. 3d). Similarly, the concentration of IL-17A, IL-17F, and GM-CSF in the serum (Fig. 3e) and the expression of Rorγt in the lymphocytes from peripheral blood (Fig. 3f) were significantly decreased. Furthermore, draining lymph node cells and splenocytes from these mice were re-stimulated in vitro, and data showed that among the CD4^+^ T cells, IL-17^+^ and GM-CSF^+^ cells were both decreased dramatically when TLR4 was deficient (Fig. 3g). Correspondingly, TLR4^−/−^ lymphocytes secreted less IL-17A, IL-17F, and GM-CSF (Fig. 3h). The data presented above collectively demonstrate that TLR4 deficiency in CD4^+^ naïve T cells can inhibit Th17 differentiation in vivo, resulting in mild EAE.

**Table 1:** EAE disease parameters

Group	Incidence	Day of onset	Peak score	Cumulative score (until day 20)
WT	93.3% (14/15)	6.0 ± 0.7	3.4 ± 0.5	26.6 ± 3.4
TLR4^−/−^	60.0% (9/15)	10.4 ± 2.0**	1.0 ± 0.4***	13.2 ± 4.6***

Data are presented as mean ± standard deviation. **P < 0.01. ***P < 0.001; Student’s *t* test

### TLR4 regulates miR-30a expression via RelA

**Fig. 4:**
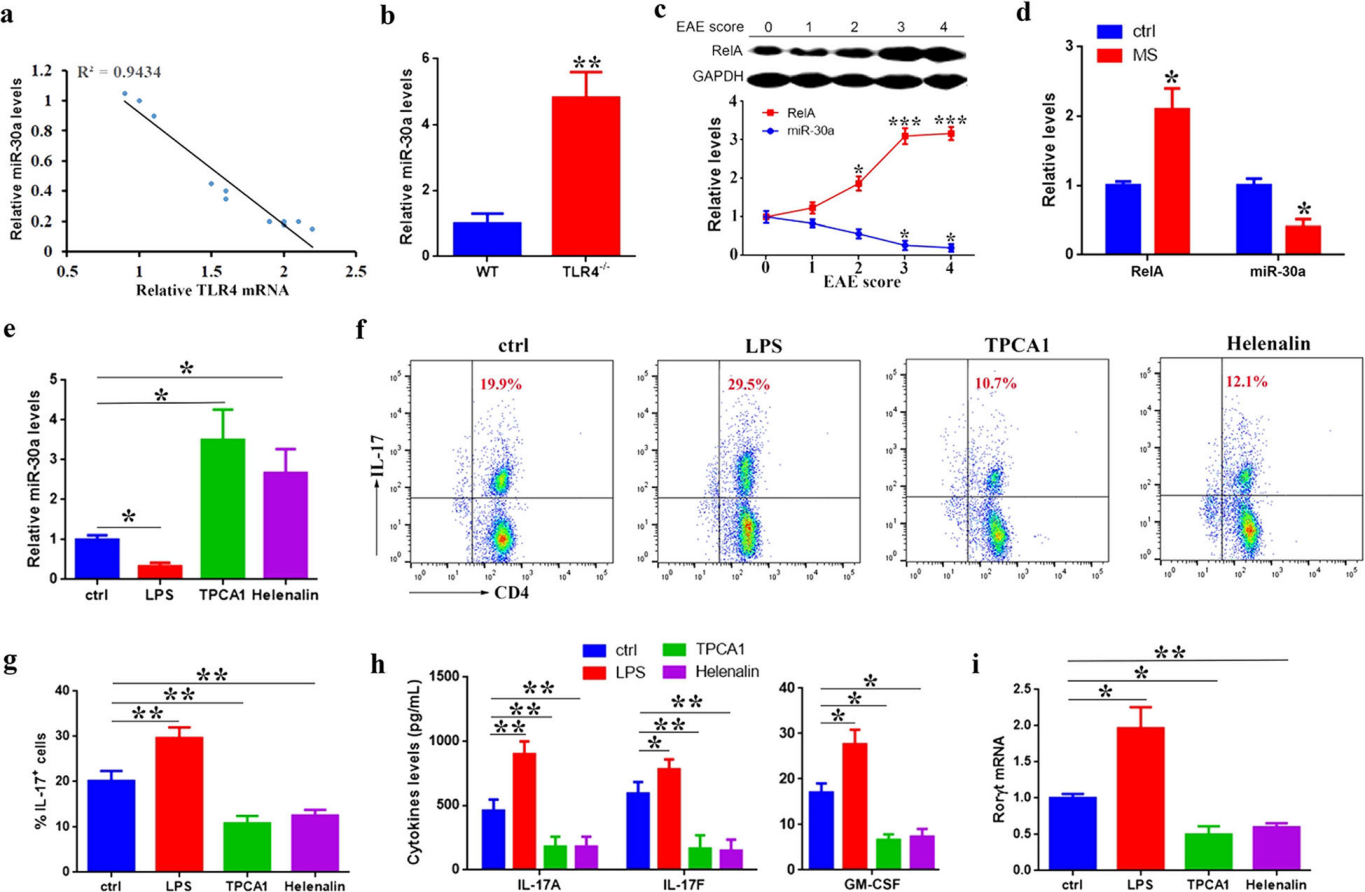
RelA regulates the expression of miR-30a. **a** Linear correlation between miR-30a and TLR4 expression at 0 h, 24 h, 48 h, and 72 h (*n* = 3 per time point, compared to 0 h) during Th17 differentiation in vitro. **b** Quantitative PCR analyses of miR-30a expression in Th17 cells from peripheral blood lymphocytes of wild type (WT) and TLR4^−/−^ mice. **c** The relative levels of RelA (Western blot) and miR-30a (quantitative PCR) in Th17 cells from peripheral blood lymphocytes of WT EAE mice at different scores (*n* = 5 per group, mice at score 0 are used as control for RelA and miR-30a respectively). **d** The relative levels of RelA (MFI analyses of flow cytometry) and miR-30a (quantitative PCR) in Th17 cells from peripheral blood lymphocytes of healthy volunteers (ctrl) and MS patients (*n* = 8 per group). **e**–**i** CD4^+^ naïve T cells from WT mice are cultured in Th17-polarizing conditions with LPS, TPCA1, or Helenalin for 3 days, then miR-30a expression in the cultured CD4^+^ cells is analyzed by quantitative PCR (**e**); percentage of stimulated IL-17^+^ cells is tested by flow cytometry (**f**, **g**); doses of IL-17A, IL-17F, and GM-CSF in culture supernatants are measured by ELISA (**h**); and Rorγt mRNA level is analyzed by quantitative PCR (**i**). CD4^+^ naïve T cells cultured in Th17-polarizing conditions are used as control (ctrl). Data are presented as mean ± standard deviation. *P < 0.05. **P < 0.01. ***P < 0.001; Student’s *t* test (**b**, **d**), one-way ANOVA (**c**, **e**, **g**–**i**). Data are representative of three experiments done in triplicate

Our previous data showed that the differentiation of Th17 could be negatively regulated by miR-30a, whose expression was decreased gradually during Th17 generation [38]. Interestingly, the miR-30a level had a negative correlation with the expression of TLR4 in the process of Th17 differentiation (Fig. 4a), even showed a significant increase in TLR4^−/−^ Th17 cells (Fig. 4b), suggesting a regulating role of TLR4 on miR-30a expression. Furthermore, we found RelA, a nuclear transcription factor that is activated by TLR4, was upregulated in correlation with increased EAE disease score, while the level of miR-30a was decreased accordingly (Fig. 4c). Similarly, compared with healthy controls, MS patients had increased RelA but decreased miR-30a (Fig. 4d).

To clarify whether TLR4 could regulate miR-30a expression via RelA, we used the LPS, a TLR4 agonist; TPCA1, an IKK-2 inhibitor [45]; and helenalin, a RelA inhibitor [46] during Th17 differentiation in vitro. The results showed that LPS treatment could downregulate the expression of miR-30a (Fig. 4e), resulting in a larger percentage of induced IL-17^+^ cells (Fig. 4f, g) with increased IL-17, GM-CSF secretion (Fig. 4h), and Rorγt expression (Fig. 4i), while inhibitor-treated CD4^+^ naïve T cells had a raised miR-30a level (Fig. 4e), lower IL-17^+^ cell percentage (Fig. 4f, g), and decreased IL-17, GM-CSF secretion (Fig. 4h), and Rorγt expression (Fig. 4i). These data show a negative regulatory role of RelA on miR-30a expression during Th17 differentiation.

### RelA recognizes specific sites in the regulatory elements of miR-30a gene

**Fig. 5:**
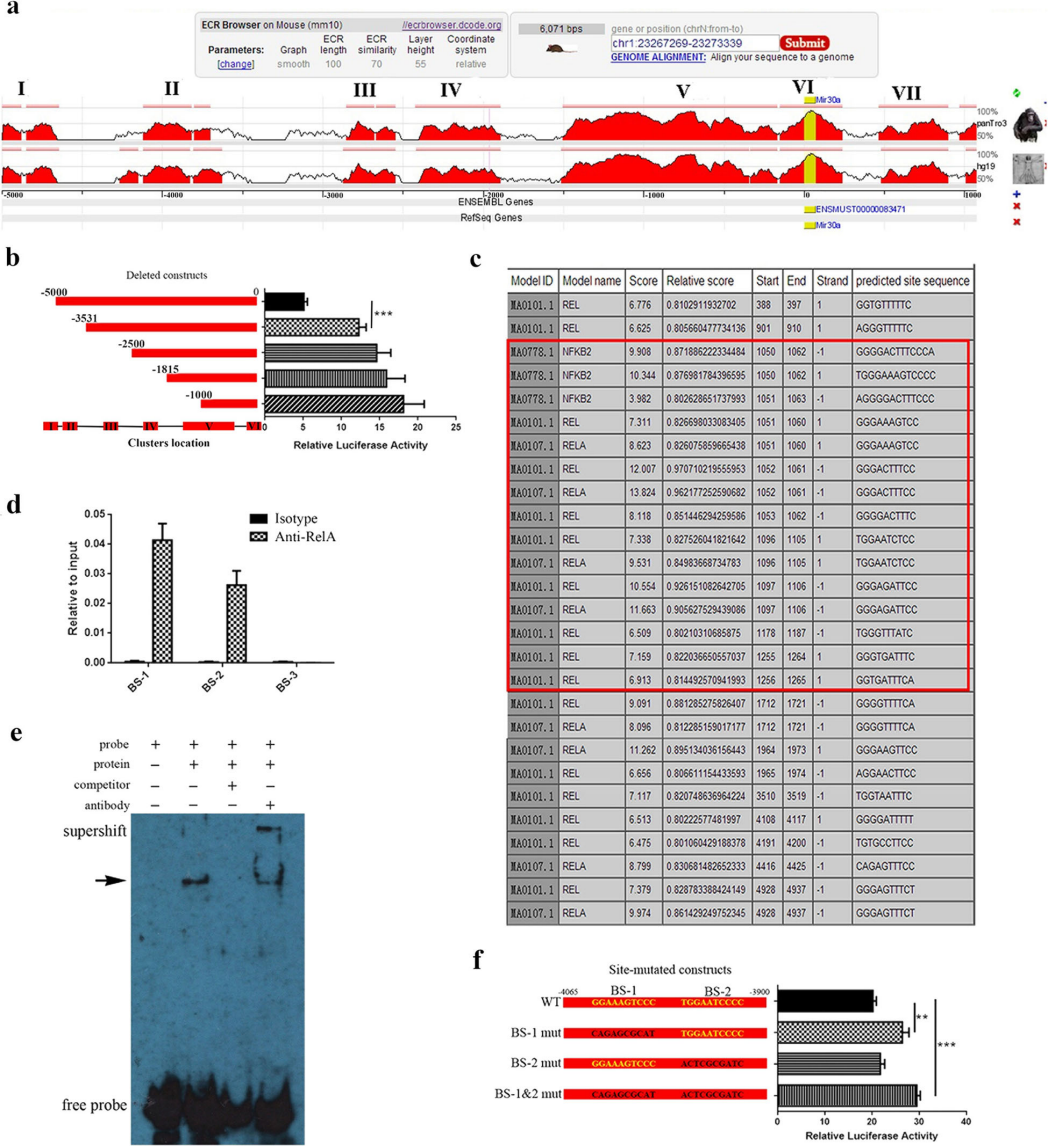
RelA binds to specific sites in the regulatory elements of miR-30a gene. **a** An expanded view of a 6-kb region (− 5000 bp~1000 bp of miR-30a gene) centered on the homologous binding sites of transcription factors among the species of mouse, human, and chimp. The region is divided into 7 parts (I–VII) according to the binding sites. Peaks represent the level of homology. **b** Binding site deletion analyses in the 5-kb up-stream region of miR-30a gene using a luciferase assay. On the left side is a schematic representation of the deleted DNA sequences carrying different clusters. The right panel shows luciferase activity normalized to Renilla luciferase activity. The construct carrying all clusters is used as control. **c** RelA binding sites in the 6-kb region. Red box shows the concentration of binding sites in cluster II. **d** The recruitment of RelA to different binding sites (BS) located in cluster II is tested by ChIP assay. The values are normalized to the input for each sample. **e** EMSA shows the combination between RelA and specific sequence. Binding complexes (arrow) are identified by supershift band with antibody against RelA. **f** Effects of binding-site mutagenesis on the RelA-mediated regulation. On the left side is a schematic representation of the normal (yellow) and site-directed altered (black) constructs. The right panel shows the luciferase activity normalized to Renilla luciferase activity. The construct with no site-directed alteration (WT) is used as control. Data are presented as mean ± standard deviation. **P < 0.01. ***P < 0.001; one-way ANOVA. Data are representative of three experiments done in triplicate

To explore the regulatory mechanism of RelA on miR-30a expression, we scanned the DNA sequence 5000 bp upstream and 1000 bp downstream of the miR-30a gene and discovered several predicted transcription factors binding site clusters (I–VII) with a high nucleotide conservation across three closely related mammals of mouse, human, and chimp (Fig. 5a). Next, different deleted DNA sequences carrying different clusters (Fig. 5b, left) were constructed for luciferase analyses, and the results revealed that deletion of cluster I and cluster II resulted in a much higher transcriptional activity than that in the construct carrying all clusters (Fig. 5b), suggesting the presence of a negative regulatory region located within cluster I and cluster II.

To explore the special transcription factors binding with cluster I and cluster II to regulate miR-30a transcription, we analyzed the promoter sequence in JASPAR and rVista 2.0 database and found several RelA binding motifs were located within the predicted transcription factor binding site clusters, especially concentrated in cluster II (Fig. 5c, Additional file 7: Figure S3). Next, we performed ChIP and EMSA experiments to determine whether RelA was able to bind directly to the putative binding sites, BS-1, BS-2, and BS-3, in cluster II (Additional file 5: Figure S1). ChIP results showed that the BS-1 and BS-2 fragments were significantly enriched after RelA immunoprecipitation, whereas no signal was observed at BS-3 (Fig. 5d, Additional file 8: Figure S4). Furthermore, in EMSA experiments, we observed that RelA bound to the BS-1 probe and this interaction disappeared by competition with an unlabeled probe (Fig. 5e). However, no specific supershift band was observed with the use of BS-2 probe (Additional file 9: Figure S5), suggesting BS-2 is not a true binding site of RelA.

To further determine the binding site of RelA in cluster II, site-mutated BS-1 and BS-2 were constructed (Fig. 5f, left). Mutations in BS-1 increased luciferase activity, while no difference was observed in constructs containing mutated BS-2 (Fig. 5f). Taken together, the above data demonstrate that RelA can modulate the expression of miR-30a via direct binding to BS-1 in cluster II.

## Discussion

Excessive differentiation and activation of Th17 is a key player in inducing inflammation in many autoimmune diseases [47–49]. However, it is still not clear how to mitigate Th17 immune response during MS and EAE to reduce pathology. Here, we explore the direct role of TLR4 activation on Th17 differentiation and find that activation of TLR4 pathway in CD4^+^ T cells promotes Th17 differentiation. Conversely, TLR4 deficiency results in decreased Th17 differentiation and mild EAE. Moreover, we demonstrate that TLR4-activated RelA can directly bind to transcriptional binding sites and negatively regulate the expression of miR-30a, a negative regulator of Th17 differentiation [38]. These results not only point out a role of TLR4 signal on Th17 differentiation in MS, but also point to a possible target for MS therapy.

Improper TLR4 activation in innate immune cells is correlated with many autoimmune diseases, including MS [50, 51]. Additionally, the increase of TLR4 in CD4^+^ T cells in MS and EAE [24, 25] provides evidence of a possible TLR4-driven regulatory mechanism in Th17 differentiation. Different from previous research using differentiated CD4^+^ T cells for transplant [25], we transfer CD4^+^ naïve T cells into Rag1^−/−^ mice for EAE induction, and results show that TLR4 deficiency in CD4^+^ naïve T cells inhibits Th17 differentiation, resulting in defective EAE disease. These results align with previous reports that TLR4 expression by CD4^+^ T cells was essential for the development of EAE [25] and that LPS directly stimulated Th17 differentiation via TLR4 pathway [19]. However, Reynolds et al. showed that LPS had no effects on promoting Th17 polarization in vitro [25]. The reasons for this discrepancy are unclear, but one possible explanation is that LPS regulates Th17 differentiation in a dose-dependent manner. In addition, CD4^+^ naïve T cells stimulated by TLR4 activation proliferate more extensively, as well as exhibit enhanced survival [25] and increased infiltration [52, 53] in comparison with inactivated cells. In the TLR4 signal pathway, NF-κB is an ubiquitous transcription factor that plays an important role in controlling the expression of genes involved in immunity and inflammation [54–56]. LPS is able to directly stimulate Th17 differentiation in vitro via phosphorylation of NF-κB1(p50) and dephosphorylation of RelB [19]. RelA transcription factor drives Th17 differentiation by directly activating Rorγt expression [57]. Moreover, knockout of myeloid differentiation factor 88 (MyD88), a key NF-κB activation factor in the TLR4 signal pathway, leads to decreased number of Th17 cells, diminished CNS inflammation, and ameliorated EAE [58]. Overall, these data provide evidences for a mechanism by which TLR4 activation directly induces Th17 differentiation.

**Fig. 6:**
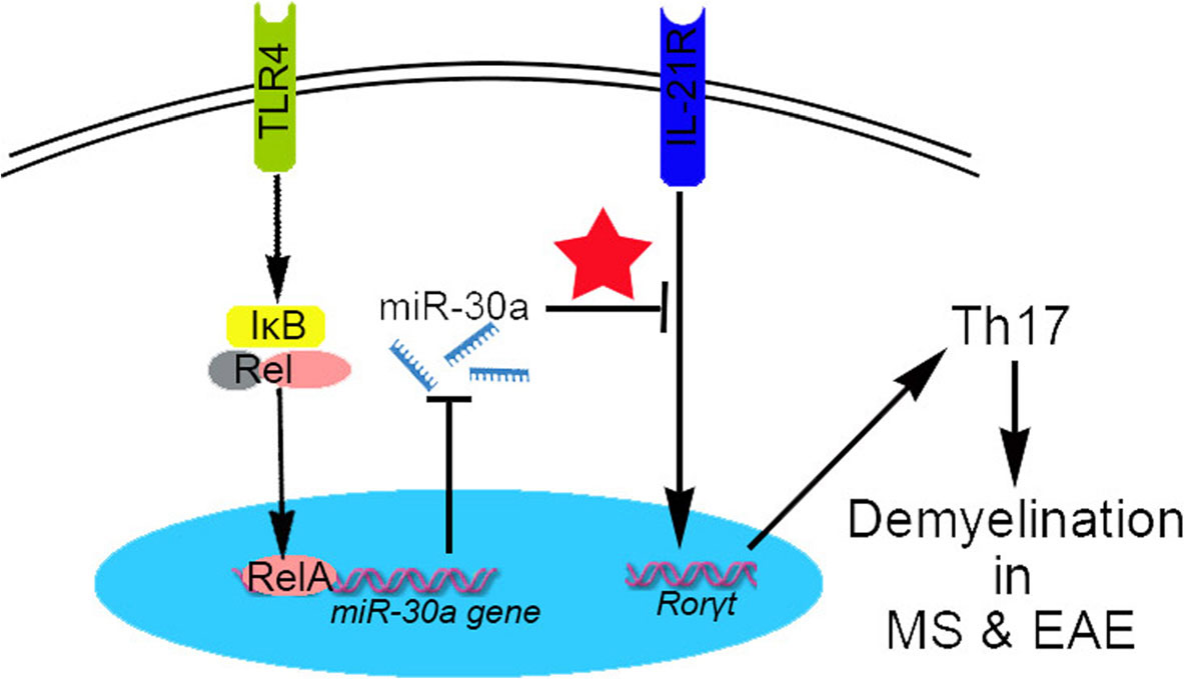
Schematic model of TLR4-RelA-miR-30a signal pathway regulating Th17 differentiation. The expression of miR-30a, a negative regulator in Th17 differentiation, is inhibited by TLR4-RelA signal. This process likely contributes to the Th17 development and demyelination in MS and EAE. Red star denotes our previous results [38]

Our previous data showed the disordered miR-30a levels in vivo contributed to Th17 ratio imbalance and EAE pathology [38]. In this paper, we further explore the reason for dysregulated expression of miR-30a. Using a series of bioinformatic analyses and experimental tests (luciferase data, ChIP, EMSA, and site-mutated construct data), we demonstrate that miR-30a is a target of RelA, which binds directly to the conserved BS-1 sequence located about 4000 bp upstream of the miR-30a gene to silence expression. In our paper, the result of ChIP identified two potential RelA binding sites, BS-1 and BS-2, while EMSA and site-mutation experiment showed no RelA binding within BS-2. One reason for this inconsistence may be that the BS-1 and BS-2 sites are too close to each other, preventing quantification as an independent event in ChIP assay. Taken together, our results demonstrate that TLR4-triggered pathway involving RelA-mediated suppression of miR-30a and its downstream target IL-21R directly regulates Th17 differentiation and EAE development (Fig. 6).

## Conclusions

Currently, several available immune inhibitor therapies for MS have achieved satisfactory effects [59, 60], but it is still difficult to fundamentally protect patients from relapsing MS. Thus, searching for more effective and more feasible therapies is the top priority for MS treatment. Here, we evaluate that (1) TLR4^−/−^ CD4^+^ naïve T cells inhibit their differentiation into Th17; (2) transfer of TLR4^−/−^ CD4^+^ naïve T cells into Rag1^−/−^ mice is defective in promoting EAE, a model of MS; and (3) TLR4-RelA-miR-30a signal pathway regulates Th17 differentiation via direct binding of RelA to the silencing element of miR-30a, an inhibitor for Th17 differentiation we previously proved. These researches not only point out the TLR4 activation as a reason for Th17 disorder in MS, but also demonstrate that modulating TLR4-RelA-miR-30a signal in Th17 may be a possible target for MS therapy.

## Supplementary information

**Additional file 1: Table S1.** Characteristics of MS patients and controls.

**Additional file 2: Table S2.** PCR primers.

**Additional file 3: Table S3.** Primers for deleted constructs.

**Additional file 4: Table S4.** EMSA probes.

**Additional file 5: Figure S1.** Pattern diagram showing the sequences and locations of primers for ChIP-PCR. The sequences in yellow show the predicted RelA binding sites (BS-1, BS-2 and BS-3).

**Additional file 6: Figure S2.** TLR4^-/-^ CD4^+^ naïve T cells transferred Rag1^-/^- mice have a less weight loss after EAE induction compared with that of wild type (WT) CD4^+^ naïve T cells transferred Rag1^-/^- mice. The ratio of body weight is normalized to initial weight of each mouse. *N* = 5 per group. Data are presented as mean ± Standard Deviation. *P < 0.05. **P < 0.01. ***P < 0.001 compared with WT group; Two-way repeated measures ANOVA. Data are representative of three experiments done in triplicate.

**Additional file 7: Figure S3.** The predicted transcription factors binding sites in cluster II of miR-30a gene by rVista online. Red boxes show the main binding sites of NF-κB.

**Additional file 8: Figure S4.** Gel electropherogram of ChIP-PCR products at three predicted RelA binding sites (BS-1, BS-2 and BS-3). M, DL2000 DNA marker.

**Additional file 9: Figure S5.** The interaction between RelA and BS-2 probe is identified by EMSA. No specific supershift band is observed when RelA antibody added.

## Data Availability

The datasets used and/or analyzed during the current study are available from the corresponding author on reasonable request.

## Abbreviations

ChIP: Chromatin immunoprecipitation
CNS: Central nervous system
EAE: Experimental autoimmune encephalomyelitis
EMSA: Electrophoretic mobility shift assay
miRNA: MicroRNA
MS: Multiple sclerosis
Th17: T helper cell 17
TLR4: Toll-like receptor 4
WT: Wild type
