# Supplementary material for: TLR4-RelA-miR-30a signal pathway regulates Th17 differentiation during experimental autoimmune encephalomyelitis development
Source: J Neuroinflammation. 2019 Sep 27;16:183. doi: 10.1186/s12974-019-1579-0 (PMC6764145; doi:10.1186/s12974-019-1579-0)
Supplement: Supplementary file 1 — Additional file 1: Table S1. Characteristics of MS patients and controls. [file 12974_2019_1579_MOESM1_ESM.docx]

Table S1. Characteristics of MS patients and controls

| Group | Sample number (Ethnic group) | Sex | Age | EDSS | Clinical stage | Disease duration (years) | Drug treatment |
| --- | --- | --- | --- | --- | --- | --- | --- |
| Control | 8 (Chinese Han) | F | 36.1±7.1 | - | - | - | - |
| RRMS | 8 (Chinese Han) | F | 39.6±8.5 | 5.1±2.2 | relapsing | 9.4±5.9 | - |

Healthy volunteers are used as control. Data are presented as mean ± Standard Deviation. “-” indicates “not applicable”. EDSS, expanded disability status scale. F, female. RRMS, relapsing remitting multiple sclerosis.
