# Supplementary material for: TLR4-RelA-miR-30a signal pathway regulates Th17 differentiation during experimental autoimmune encephalomyelitis development
Source: J Neuroinflammation. 2019 Sep 27;16:183. doi: 10.1186/s12974-019-1579-0 (PMC6764145; doi:10.1186/s12974-019-1579-0)
Supplement: Supplementary file 2 — Additional file 2: Table S2. PCR primers. [file 12974_2019_1579_MOESM2_ESM.docx]

Table S2. PCR primers

| Name | Forward (5’-3’) | Reverse (5’-3’) |
| --- | --- | --- |
| Rorγt | TGCAAGACTCATCGACAAGG | AGGGGATTCAACATCAGTGC |
| TLR4 | AAACTTGCCTTCAAAACCTGGC | ACCTGAACTCATCAATGGTCACATC |
| β-actin | GAGACCTTCAACACCCCAGCC | AATGTCACGCACGATTTCCC |
