# Supplementary material for: TLR4-RelA-miR-30a signal pathway regulates Th17 differentiation during experimental autoimmune encephalomyelitis development
Source: J Neuroinflammation. 2019 Sep 27;16:183. doi: 10.1186/s12974-019-1579-0 (PMC6764145; doi:10.1186/s12974-019-1579-0)
Supplement: Supplementary file 3 — Additional file 3: Table S3. Primers for deleted constructs. [file 12974_2019_1579_MOESM3_ESM.docx]

Table S3. Primers for deleted constructs

| Location | Forward (5’-3’) | Reverse (5’-3’) |
| --- | --- | --- |
| -5000~-1 | CATGGGTACCAGGTGCCAGGGAACTGCTGA | CATGCTCGAGTCACTGTCAACAGCAATATACC |
| -3531~-1 | CATGGGTACCTCAGAATAGAAAAGTGAGTAACG | CATGCTCGAGTCACTGTCAACAGCAATATACC |
| -2500~-1 | CATGGGTACCCCTTGAATTGGACTAAAAGAGTG | CATGCTCGAGTCACTGTCAACAGCAATATACC |
| -1815~-1 | CATGGGTACCGCAAGATATGAGCTAGGTGA | CATGCTCGAGTCACTGTCAACAGCAATATACC |
| -1000~-1 | CATGGGTACCTGTTAGTTTCAGTAAACATGATGC | CATGCTCGAGTCACTGTCAACAGCAATATACC |

The sequences in green show the restriction sites of *Kpn*I (GGTACC) and *Xho*I (CTCGAG).
