# Supplementary material for: TLR4-RelA-miR-30a signal pathway regulates Th17 differentiation during experimental autoimmune encephalomyelitis development
Source: J Neuroinflammation. 2019 Sep 27;16:183. doi: 10.1186/s12974-019-1579-0 (PMC6764145; doi:10.1186/s12974-019-1579-0)
Supplement: Supplementary file 4 — Additional file 4: Table S4. EMSA probes. [file 12974_2019_1579_MOESM4_ESM.docx]

Table S4. EMSA probes

| Sites | Sequences (5’-3’) |
| --- | --- |
| S1 | GGAGCAGGATGGGAGTGGGGTGGAAAGTCCCTTATTAGCCTGGAGGCTGG |
| S2 | CTGGGATGCCCTGACCCTCTGGAATCCCCCTCCCTTGTAAGAATGCCAGCCA |

The sequences in blue show the predicted binding sites of RelA.
