# Supplementary figures and images for: TLR4-RelA-miR-30a signal pathway regulates Th17 differentiation during experimental autoimmune encephalomyelitis development
Source: J Neuroinflammation. 2019 Sep 27;16:183. doi: 10.1186/s12974-019-1579-0 (PMC6764145; doi:10.1186/s12974-019-1579-0)

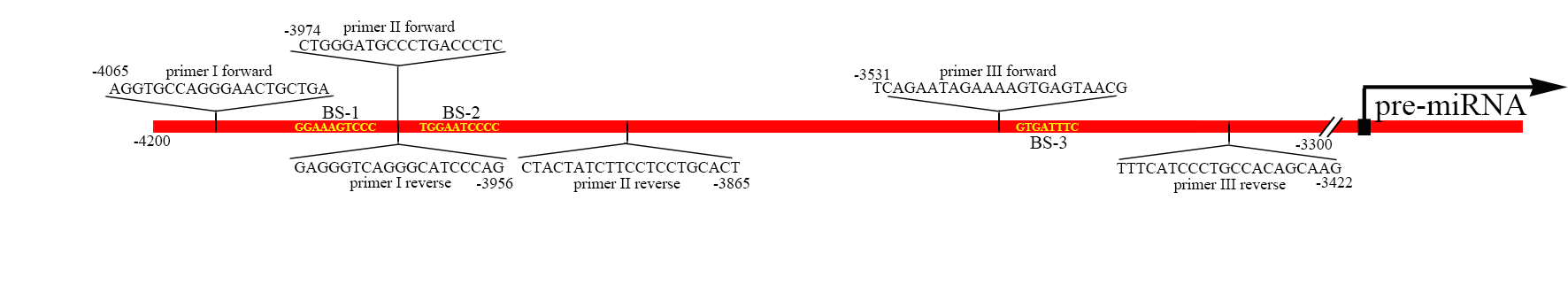

Supplement: Supplementary file 5 — Additional file 5: Figure S1. Pattern diagram showing the sequences and locations of primers for ChIP-PCR. The sequences in yellow show the predicted RelA binding sites (BS-1, BS-2 and BS-3). [file 12974_2019_1579_MOESM5_ESM.tif]

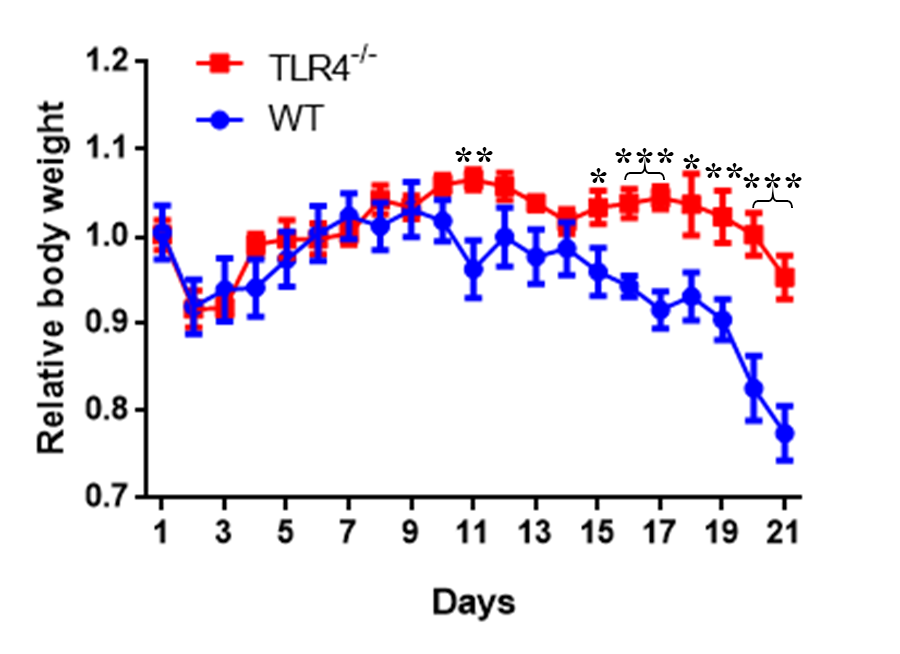

Supplement: Supplementary file 6 — Additional file 6: Figure S2. TLR4-/- CD4+ naïve T cells transferred Rag1-/- mice have a less weight loss after EAE induction compared with that of wild type (WT) CD4+ naïve T cells transferred Rag1-/- mice. The ratio of body weight is normalized to initial weight of each mouse. N = 5 per group. Data are presented as mean ± Standard Deviation. *P < 0.05. **P < 0.01. ***P < 0.001 compared with WT group; Two-way repeated measures ANOVA. Data are representative of three experiments done in triplicate. [file 12974_2019_1579_MOESM6_ESM.tif]

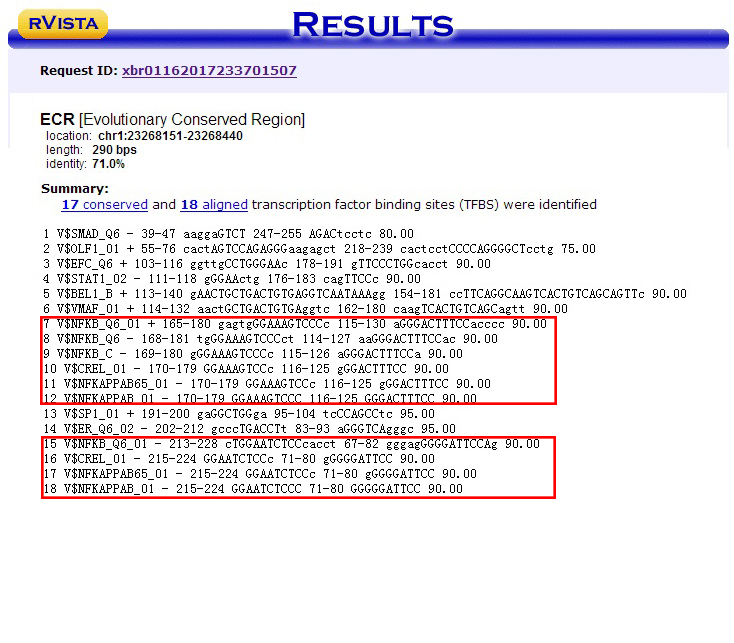

Supplement: Supplementary file 7 — Additional file 7: Figure S3. The predicted transcription factors binding sites in cluster II of miR-30a gene by rVista online. Red boxes show the main binding sites of NF-κB. [file 12974_2019_1579_MOESM7_ESM.jpg]

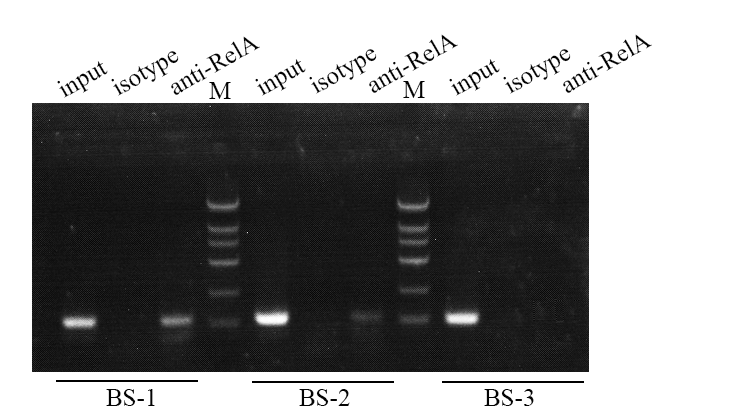

Supplement: Supplementary file 8 — Additional file 8: Figure S4. Gel electropherogram of ChIP-PCR products at three predicted RelA binding sites (BS-1, BS-2 and BS-3). M, DL2000 DNA marker. [file 12974_2019_1579_MOESM8_ESM.tif]

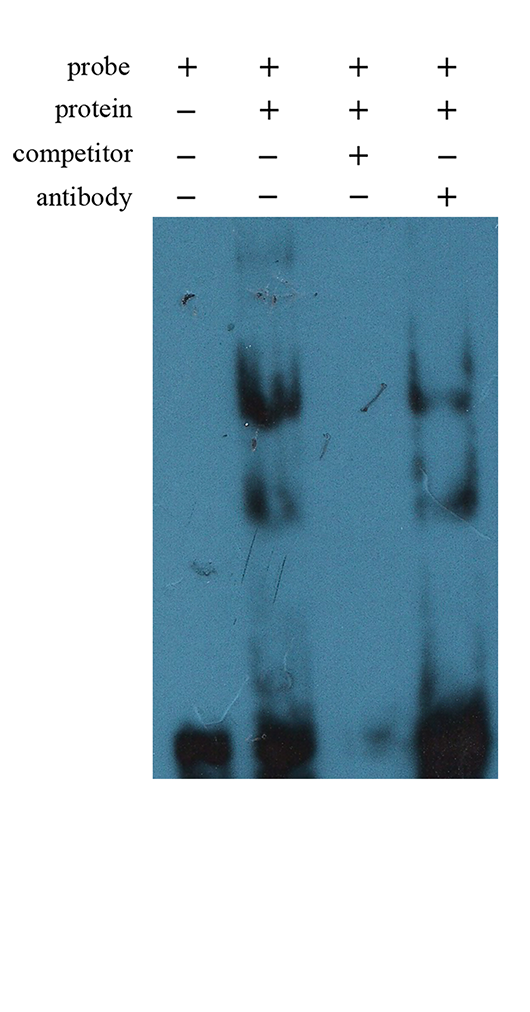

Supplement: Supplementary file 9 — Additional file 9: Figure S5. The interaction between RelA and BS-2 probe is identified by EMSA. No specific supershift band is observed when RelA antibody added. [file 12974_2019_1579_MOESM9_ESM.tif]
